# Tackling heart failure in sub-Saharan Africa: the imperious need for hypertension prevention and control

**DOI:** 10.11604/pamj.2020.36.372.24528

**Published:** 2020-08-31

**Authors:** Jean Jacques Noubiap

**Affiliations:** 1 Centre for Heart Rhythm Disorders, University of Adelaide and Royal Adelaide Hospital, Adelaide, Australia

**Keywords:** Heart failure, hypertension, cardiovascular disease, Africa

## Editorial

Heart failure (HF) is a leading contributor to the global burden of disease, affecting about 26 million people worldwide [1]. It is associated with high morbidity, recurrent and prolonged hospitalization, poor quality of life, premature death and massive economic loss [1, 2]. Sub-Saharan African (SSA) populations are disproportionally affected. According to the International Congestive Heart Failure (INTER-CHF) prospective cohort study, patients with HF in sub-Saharan Africa (SSA) have the highest mortality rate, with a third of them who die within a year [2]. The excess HF-related mortality in the region is mostly attributed to weak health care systems that are already overwhelmed with the burden of infectious diseases, and to poor access to guideline-directed medical treatment [2, 3]. Furthermore, HF in SSA occurs at a much younger age, mostly in professionally active adults, leading to significant loss of economic productivity [4-6].

Largely unknown about two decades ago, the epidemiology of HF in SSA has been increasingly studied in the recent years. The sub-Saharan Africa Survey of Heart Failure (THESUS-HF) was pioneer to prospectively investigate the causes, clinical features, treatment, and outcomes of patients admitted with acute HF in academic hospitals in SSA [7]. THESUS-HF along with several other reports have revealed that, in a stark contrast with data from most European and North American populations, HF in SSA populations is predominantly non-ischemic and most commonly due to hypertension. This is corroborated by the findings from the study by Mandi *et al*. published in this volume of the Pan African Medical Journal [8].

Mandi *et al*. conducted a prospective cohort study at the Regional Hospital Center of Tenkodogo, a tertiary hospital in the eastern region of Burkina Faso. They found that 17.6% of patients who attended the cardiology unit had acute HF, mostly de novo cases (80.2%). Importantly, hypertensive heart disease accounted for up to half of all cases of HF. Other key information is the young age of patients, with an average of 58 years; the high mortality of up to 31 per 100 patient-years of follow-up; a strikingly low use of beta-blockers (18.8%) that are pivotal in guideline-directed medical treatment of HF; and a high frequency of atrial fibrillation (29.5%), one of the highest reported in HF patients in the region [9].

The most salient message from the study by Mandi *et al*. although not surprising, is the high burden of hypertensive heart disease. This aligns with a recent comprehensive review of the epidemiology of HF in SSA which showed that four in ten cases of HF in the region are directly attributed to hypertension [4]. This reminds us about the ongoing disaster caused by hypertension in SSA. Indeed, unlike many countries in other parts of the globe where the prevalence of hypertension is steadily decreasing, in SSA countries, this prevalence is rising [10]. It is estimated that more than 30% of adults in SSA have hypertension, compared to less than 20% about 30 years ago [10, 11]. More worrisome, this high prevalence of hypertension is associated with low awareness, treatment and control rates, with only 27% of affected people who are aware of their condition, 18% who are on treatment, and 7% who achieve controlled blood pressure (BP) in the region [11]. As a result, in 2017, hypertension was a leading risk factor for death and disability, accounting for ~580,000 deaths and ~14 million disability-adjusted life years in SSA [12].

The surge in hypertension prevalence in SSA over the last two decades is part of the epidemiologic transition that is occurring in region, driven by socioeconomic and cultural changes characterized by reduced physical activity, elevated stress, unhealthy dietary patterns with high saturated fat and salt consumption, and alcohol abuse [13]. All these environmental factors play on a permissive genetic background that predisposes African populations to be more sensitive to salt, i.e. to be more susceptible to sodium-related increase in blood pressure [14]. The major contribution of modifiable environmental factors on the incidence of hypertension suggests that it can be prevented to a large extent. Furthermore, considering the huge economic burden imposed by hypertension on health care systems and individuals in SSA, with an estimated 7.3% of total health care spending directly related to hypertension and its complications, prevention of hypertension should be an imperious priority in the region [15].

The prevention of hypertension should be integrated in an umbrella of interventions for primordial prevention of cardiovascular disease in general. This starts with educating populations on hypertension and other cardiovascular risk factors, and measures to prevent them. Population-wide interventions should be implemented to promote healthy diet, by reducing saturated and trans fat, sugar and salt intake, by increasing potassium and fruits consumption [16]; and by encouraging physical activity and a healthy living and working environment. The food industry should contribute to these efforts by selling healthy products, within a well-established legislation framework to mandate food labelling and compliance to recommended levels of dietary salt and sugar.

The next step is to scale up hypertension screening and effective management, especially in the context of unacceptably high rates of undiagnosed, untreated and poorly controlled disease in SSA [11]. Systematic blood pressure measurement is pivotal to ensure that no adult who encounters a health facility misses the opportunity to be screened for hypertension, but bearing in mind the significant proportion of white coat hypertension [17]. Community blood pressure screening should also be encouraged through the provision of blood pressure devices at the workplace and churches, for instance. Regarding treatment, approaches to achieve a global cardiovascular risk reduction are the most effective [18]. Hence, a cardiovascular risk assessment should be performed, looking for other potential risk factors such as obesity, diabetes and dyslipidaemia, and target organ damage including left ventricular dysfunction, cerebrovascular disease and renal dysfunction that should be comprehensively addressed. It is essential that good quality generic cardiovascular medications are provided to patients at low cost, and ultimately, that universal health coverage is implemented to ensure that the large majority of the population have access to effective health care [18]. Home blood pressure monitoring (HBPM) should be scaled up to improve blood pressure control. When combined with appropriate patient education, lifestyle modification and adherence to medications, HBPM stands out as a valuable tool in the treatment of hypertension [19]. Furthermore, health care workers should be trained to appropriately manage hypertension, and be provided with contextualized national guidelines for the diagnosis and treatment of cardiovascular risk factors and diseases. Task sharing or shifting strategies should be considered for implementation at the primary care level to enhance access to health care [19].

All these strategic components for the prevention and control of hypertension require a constant implication and a strong collaboration between national governments and health organizations, international agencies and scientific societies such as the World Health Organization (WHO), the World Heart Federation and the International Society of Hypertension, and international aid and health funding bodies. In May 2013, the 66^th^World Health Assembly endorsed the WHO Global Action Plan for the Prevention and Control of Noncommunicable diseases 2013-2020. This global initiative provides a road map and a menu of policy options to be implemented collectively between 2013 and 2020 by WHO Member States and various organizations, in order to achieve nine global targets including a 30% reduction in mean population salt intake; a 25% reduction in the prevalence of raised blood pressure; and a 25% relative reduction in premature mortality from noncommunicable diseases by 2025. Considering the current trends in the burden of hypertension and other noncommunicable diseases [12], it is very unlikely that these targets will be attained in SSA by 2025. However, SSA countries should build on the progress made and capitalize the experience gained to develop and deploy more feasible and effective local policies and plans to curb the burden of hypertension and other noncommunicable diseases.

**Disclosures:** Dr Noubiap is supported by a Postgraduate Scholarship from the University of Adelaide.
